# Evaluation of the effect of ambrisentan on digital microvascular flow in patients with systemic sclerosis using laser Doppler perfusion imaging: a 12-week randomized double-blind placebo controlled trial

**DOI:** 10.1186/s13075-015-0558-9

**Published:** 2015-03-05

**Authors:** Nilanjana Bose, James Bena, Soumya Chatterjee

**Affiliations:** Hunter Holmes McGuire VA Medical Center, Richmond, VA USA; Department of Rheumatic and Immunologic Diseases, Orthopedics and Rheumatology Institute, Cleveland Clinic, 9500 Euclid Avenue, Desk A50, Cleveland, OH 44195 USA

## Abstract

**Introduction:**

In patients with systemic sclerosis (SSc), digital ischemia results from an occlusive microvasculopathy that may not respond adequately to conventional vasodilators. Endothelin receptor antagonists can potentially modify the fibroproliferative vascular remodeling in SSc, and hence their use may be justified in the management of digital ischemia. The objective of this clinical trial was to evaluate the effect of ambrisentan, a selective endothelin type A receptor antagonist, on microvascular blood flow in patients with limited systemic sclerosis (SSc) using laser Doppler perfusion imaging (LDPI).

**Methods:**

In this randomized, double-blind, placebo controlled study we enrolled 20 patients with limited SSc. Fifteen patients received ambrisentan 5 mg daily for one month and then 10 mg daily for two months, and five received a placebo. There were three visits: weeks 0 (baseline), one and 12. Three patient-oriented questionnaires were completed at each visit: Scleroderma-Health Assessment Questionnaire (S-HAQ), Raynaud Condition Score (RCS), and Pain-Visual Analog Scale (P-VAS). At each visit, LDPI was used to obtain three blood flow readings involving regions of interest in second to fifth fingers of the non-dominant hand at room temperature (25°C) and after cooling (10°C) for two minutes.

**Results:**

There were 16 females (80%); mean age was 50 years. None of the differences in blood flow (as measured by LDPI) were significant both at baseline and after cooling. However, patients in the ambrisentan group showed significant improvement in the patient-oriented outcomes: RCS (P = 0.001) and S-HAQ score (P = 0.005).

**Conclusions:**

This pilot study did not show evidence of significant increase in digital blood flow over time; however, there was an improvement in RCS and S-HAQ score. We conclude that continuous use of ambrisentan for three months does not seem to significantly improve digital blood flow in SSc patients.

**Trial registration:**

Clinicaltrials.gov NCT01072669. Registered 19 February 2010.

**Electronic supplementary material:**

The online version of this article (doi:10.1186/s13075-015-0558-9) contains supplementary material, which is available to authorized users.

## Introduction

Raynaud phenomenon (RP) affects 90% to 95% of patients with systemic sclerosis (scleroderma, SSc), resulting in digital ulcers in approximately 30% of patients annually [1-3]. The proliferative microvasculopathy leading to digital ischemia not only results in hypoxemic tissue damage, but it also initiates fibroblast activation and promotes tissue fibrosis.

Endothelial injury is thought to precede loss of normal vasodilator response to nitric oxide and prostacyclin, leading to abnormal responses to vasoconstrictive mediators including endothelin-1 (ET-1) and catecholamines [4]. Serum ET-1 level is increased in SSc patients [5,6] making it a rational therapeutic target in this disease [7].

The use of endothelin receptor antagonists may be justified in SSc patients with digital ischemia refractory to conventional vasodilators, since in addition to their vasodilator properties, these drugs also have favorable effects on fibro-proliferative vascular remodeling in the long term. It has been shown that small doses of bosentan, a dual endothelin receptor antagonist, improve endothelial function in SSc patients without altering hemodynamic parameters [8]. This would support a direct, reversible effect of endothelin in SSc associated vasculopathy.

Assessment and documentation of the extent and severity of digital microvascular involvement in SSc have been challenging, and have been traditionally based on clinical impressions [9]. In addition, interpretation of therapeutic responses in RP has also typically been based on highly subjective patient-reported responses. Lately, laser Doppler perfusion imaging (LDPI) has gained acceptance as an emerging technology that allows objective measurement of cutaneous blood flow in RP. Prior studies including our own validated that LDPI reliably evaluates digital microvascular flow in SSc [10-18].

The Doppler principle has been utilized by LDPI technology to calculate skin perfusion, since the magnitude and frequency distribution of the Doppler shifted light are directly related to the number and velocity of moving blood cells. LDPI involves perfusion mapping of areas, rather than examination of blood flow at a single point as with laser Doppler flowmetry [19]. Cutaneous perfusion is unaffected, as there is no physical contact with skin, and dyes or tracer elements are not required. Measuring blood flow in adjacent points within an image and performing averaging techniques overcomes the problem with point to point cutaneous blood flow heterogeneity, and provides clinically meaningful evaluation of perfusion. It also allows more precise statistical assessment of change in blood flow with pharmacologic intervention.

ET-1 antagonists are effective in pulmonary arterial hypertension (PAH) in SSc and, hence, received regulatory approval for this indication. In addition, bosentan has been shown to decrease the occurrence of new digital ulcers in patients with SSc [2,9].

Ambrisentan selectively blocks the vasoconstrictive effect of ET_A_ receptor stimulation, leaving the vasodilatory effect of ET_B_ receptor stimulation unopposed. It has demonstrated a significant improvement in exercise capacity (six-minute walk) along with clinical improvement in patients with idiopathic and SSc-associated PAH with World Health Organization (WHO) functional class II and III symptoms [20]. A small prospective open-label study (20 patients) demonstrated that ambrisentan may also be useful in reducing ulcer burden and healing ischemic digital ulcers in SSc [21]. These studies led us to hypothesize that ambrisentan increases digital micro-vascular flow (as measured by LDPI) in patients with RP secondary to SSc.

## Methods

### Study design

**Figure 1:**
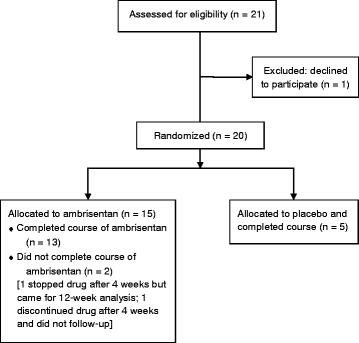
**Trial profile.**

We conducted a randomized, double-blind, placebo controlled trial (ClinicalTrials.gov identifier: NCT01072669) to evaluate the effect of ambrisentan on digital micro-vascular blood flow in patients with limited SSc over a three-month period. Subjects were recruited from the Cleveland Clinic SSc database. Twenty patients were randomized to ambrisentan or placebo in a 3:1 ratio and each patient was treated for a total period of three months (Figure 1). The study was approved by the institutional review board of the Cleveland Clinic, Cleveland, Ohio, U.S.A.

### Subjects

Subjects had limited SSc with disease duration <8 years and satisfied the American College of Rheumatology 1980 criteria for diagnosis [1,22]. Informed consents were obtained from all patients prior to participation in the study. Only patients with limited SSc were included, in order to have a fairly homogeneous group of patients with chronic digital ischemia, who are less likely to drop out during the course of the study because of major internal organ complications of SSc. RP was defined as episodic, bilateral, digital color changes (at least two out of three possible phases: pallor, cyanosis, rubor), provoked by cold exposure or by emotional stress. Subjects were ≥18 years of age and were able to give informed consent. Patients with any of the following conditions were excluded: (1) active digital ulcers or prior history of digital ulcers with resultant scarring or significant pitting of digits in the regions of interest (ROI), (2) pregnancy (ambrisentan is pregnancy category X) or inability to consistently use two reliable forms of contraception during the study, (3) moderate to severe hepatic dysfunction, (4) hemoglobin <10% below the lower limit of normal, (5) advanced cardiopulmonary disease, (6) inability to discontinue conventional vasodilator treatment for RP at least one week prior to study initiation, (7) PAH, (8) concomitant use of medications that are known to interact with ambrisentan [23] and (9) current tobacco use.

### Treatment and evaluation protocol

Patients underwent a physical examination at baseline along with measurement of hemoglobin level, liver function (alanine aminotransferase (ALT), aspartate aminotransferase (AST) and bilirubin), and urine or serum pregnancy test [20,23]. Women of reproductive age had to use two reliable forms of contraception along with appropriate counseling, unless they had tubal ligation or an intrauterine device (IUD) [23]. Subjects had to abstain from caffeine and other vasoactive agents, at least two days prior to randomization and throughout the study period. Patients also stopped concurrent medications for RP one week prior to initiation of study drug/placebo and had to stay off these medications throughout the study period. Storage, dispensing and randomization of ambrisentan and the matching placebo were conducted under the supervision of our institutional research pharmacy. Investigators and patients were blinded to the treatment.

After baseline evaluation, patients were randomized to ambrisentan or placebo and underwent three visits at weeks 0, 1 and 12. Subjects received ambrisentan 5 mg orally daily or identical placebo for the first four weeks and then were up-titrated to ambrisentan 10 mg daily or placebo for the rest of the study. Adherence to treatment was assessed by pill counting at each follow-up visit.

The non-dominant hand was used for scanning unless precluded by digital ulcers, skin infections, injury, surgical scars, or amputations. The index, middle, ring and little fingers were designated as regions 1, 2, 3 and 4, respectively. The region of worst perfusion was also selected for each patient and evaluated over the study period. All study measurements were taken in a quiet room, with subjects seated comfortably with their non-dominant forearm resting on a table at heart level.

At each visit, three baseline blood flow readings were recorded at room temperature (25°C), and three after cooling of the fingers (10°C for two minutes, with the help of a cooling flask). The cooling flask was a 1,000 ml spherical glass flask that contained ice and water, the surface temperature of which was maintained at 10°C with a thermometer and a stirrer. Patients placed their hand around the flask for two minutes to achieve cooling of the fingers to 10°C, before each of the three observations at cold temperature. Regions of interest (ROI) over the dorsum of the second to fifth fingers distal to the distal inter-phalangeal joints were selected, and the mean of each of these three sets of readings was obtained. At each visit, patients also completed three questionnaires: the Scleroderma-Health Assessment Questionnaire (S-HAQ) [24], Raynaud Condition Score (RCS) [25], and Pain-Visual Analogue Scale (P-VAS) [26] (Additional files 1, 2 and 3).

### Safety monitoring

Patients were carefully monitored for adverse events. Due to the hepatotoxic potential of ambrisentan, monthly liver function tests were performed during the study period. Study treatment was to be withheld if hepatic transaminases were elevated to >3 times the upper limit of normal (ULN) or bilirubin to >2 times the ULN; if levels did not return to normal, treatment was to be permanently discontinued. Hemoglobin levels were also checked at one and three months. For female patients, a serum or urine pregnancy test was checked once a month, unless they were post-menopausal and/or had a history of IUD placement, sterilization or hysterectomy. Counseling for women of childbearing potential (n = 3) regarding consistent use of two reliable forms of contraception was reinforced at each visit. These women were advised against pregnancy for at least one month after completion of the study.

### Laser Doppler perfusion imaging (LDPI) technology

The Perimed PeriScan PIM II Laser Doppler Perfusion Imager uses a low power laser beam operating at 670 nm wavelength with a maximum depth of 0.6 mm and average depth of 0.3 mm [27]. The imager targets the Doppler signal to the skin surface. Backscattered light from the skin surface is registered by a photo detector in the scanner head that lies about 30 cm above the tissue surface. The reading is then reported to an integrated software system (LDPIwin®) that processes the signal and performs statistical calculations [11,19]. Perfusion signals appear as two-dimensional color coded images (flow-maps of the spatial distribution of tissue perfusion) with a scale ranging from dark blue (lowest value) to red (highest value). Blood flow measurement by LDPI is commonly referred to as ‘flux’ and expressed in perfusion units (pU) [27].

### Statistical methods

#### Sample size analyses

Sample size calculations were performed based on data from our previous pilot study on SSc patients [11], where mean perfusion under normal conditions for SSc patients was 1.05 pU, with a standard deviation (SD) of 0.40 pU. With a 3:1 randomization scheme and using a SD of 0.40, with 20 patients, it was calculated that there would be at least 80% power to detect differences in the change from baseline between groups of at least 0.65 units and 0.45 units, under the assumptions of moderate (r = 0.50) and strong (r = 0.75) correlation between time points, respectively. These power calculations were performed using SAS software (version 9.1, Cary, NC, USA).

#### Randomization

A blocked randomization scheme was used since seasonal differences may exist in symptom severity. The randomization list was provided by the Quantitative Health Sciences Department of our institution. Analyses were performed using the intention-to-treat principle. Clinical and demographic factors including age, race, gender, and disease duration were collected for each subject, and summarized using means and standard deviations for continuous measures and frequencies and percentages for categorical factors. To assess differences in perfusion levels over time, the mean perfusion across all four finger ROIs was calculated for each scan. ROI perfusion levels were calculated using unweighted, variance weighted, and site weighted means. Results were very similar, so for brevity, only unweighted means are reported here.

Repeated measure analysis of variance models were then fitted using a compound symmetry correlation structure. These models used all three time points and allowed evaluation of overall differences across time as well as paired differences between time points. In each model, the survey or ROI response was used as the response, while time, treatment and their interaction were used as predictors. Models for ROI were fitted separately for normal and cold conditions because the correlation pattern differed across conditions. Statistical modeling was performed using the Mixed Procedure with SAS software (version 9.1, Cary, NC, USA). All hypothesis tests used a 0.05 significance level. No corrections for multiple comparisons were performed.

Study data were collected and managed using REDCap electronic data capture tools [28]. REDCap (Research Electronic Data Capture) is a secure, web-based application designed to support data capture for research studies, providing an intuitive interface for validated data entry and automated export procedures for seamless data downloads to common statistical packages.

#### Outcomes

We expected blood flow changes at one week to reflect treatment effect (if any) related to vasodilatation and at three months to the anti-fibro-proliferative effects of ambrisentan. Therefore, the primary outcome measures were the mean change of blood flow at one week and at twelve weeks of treatment. Secondary outcome measures included changes in S-HAQ scores, RCS, P-VAS scores, progression or regression of digital ulcers outside the ROI, and safety and tolerability.

## Results

**Figure 2:**
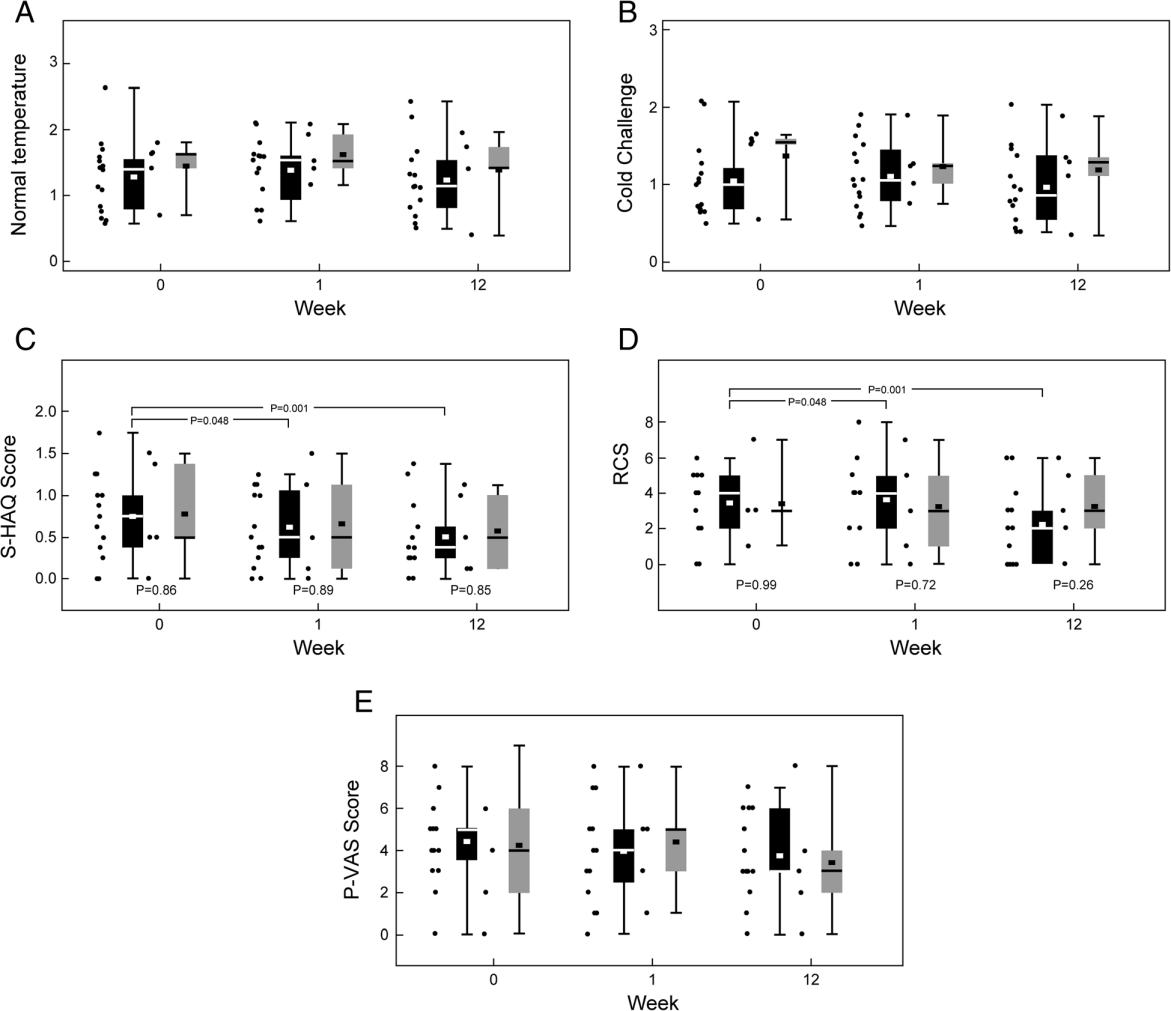
**Boxplots showing perfusion measures at normal temperature and during cold challenge, S-HAQ scores, RCS and P-VAS scores. (A)** Boxplots showing perfusion measures at baseline, week 1 and week 12 at normal temperature. Black boxes represent ambrisentan and gray boxes represent placebo. The bottom and top of the boxes represent the 25th and 75th percentiles, respectively; the band within the box is the median, and the ends of the whiskers are the minimum and maximum of all the data in that group; the square within the box is the mean. The dots on the left of each boxplot represent perfusion measures from individual subjects. **(B)** Boxplots showing perfusion measures at baseline, week 1 and week 12 during cold challenge. **(C)** Boxplots showing S-HAQ scores at baseline, week 1 and week 12. **(D)** Boxplots showing RCS at baseline, week 1 and week 12. **(E)** Boxplots showing P-VAS scores at baseline, week 1 and week 12. P-VAS, Pain-Visual Analog Score; RCS, Raynaud Condition Scale; s-HAQ, Scloderma-Health Assessment Questionnaire.

Out of 21 patients screened, 20 were included in the study; 15 were assigned to ambrisentan and 5 to placebo (Figure 1). The two groups were well matched with respect to demographic features, baseline disease characteristics and prior therapy (Table 1). Sixteen patients were women (80%) and 15 were white (75%). The mean age was 50 years (range: 20 to 70 years). Figure 2 shows boxplots of the perfusion measures (A and B) and the three survey scores (C, D, and E). Tables 2 and 3 show the ‘within group’ changes seen in the repeated measure analyses of variance models.

**Table 1:** **Summary of the demographic characteristics of the cohort**

		**Total**	**Ambrisentan**		**Placebo**	
**Factor**	**Level**	**Number**	**Number**	**(%)**	**Number**	**(%)**
Gender						
	Female	16	13	86.7	3	60.0
	Male	4	2	13.3	2	40.0
Age						
	Mean (SD)	20	15	50.6 (12.8)	5	46.8 (10.5)
Race						
	Non-White	5	4	26.7	1	20.0
	White	15	11	73.3	4	80.0
Ethnicity						
	Non-Hispanic	20	15	100.0	5	100.0
Smoking						
	No	20	15	100.0	5	100.0
Scleroderma						
	Limited	20	15	100.0	5	100.0

SD = standard deviation.

**Table 2:** **Summary of changes of all region perfusion responses by treatment and time under normal conditions**

		**Placebo**			**Ambrisentan**		
**Measure**	**Change period**	**Change (95% CI)**	**Change** ***P*** **-value**	**Overall** ***P*** **-value**	**Change (95% CI)**	**Change** ***P*** **-value**	**Overall** ***P*** **-value**
All	1 W - Base	0.18 (−0.27,0.63)	0.42	0.54	0.11 (−0.15,0.37)	0.41	0.61
	12 W - Base	−0.06 (−0.51,0.39)	0.80		−0.01 (−0.28,0.25)	0.93	
	12 W - 1 W	−0.24 (−0.69,0.21)	0.29		−0.12 (−0.38,0.15)	0.37	
R1	1 W - Base	0.23 (−0.18,0.64)	0.26	0.29	0.12 (−0.11,0.36)	0.29	0.30
	12 W - Base	−0.08 (−0.48,0.33)	0.71		−0.06 (−0.30,0.18)	0.62	
	12 W - 1 W	−0.31 (−0.71,0.10)	0.14		−0.18 (−0.42,0.06)	0.13	
R2	1 W - Base	0.15 (−0.32,0.62)	0.52	0.76	0.03 (−0.24,0.31)	0.80	0.80
	12 W - Base	0.00 (−0.47,0.47)	0.99		−0.06 (−0.34,0.22)	0.68	
	12 W - 1 W	−0.15 (−0.62,0.32)	0.53		−0.09 (−0.37,0.19)	0.51	
R3	1 W - Base	0.08 (−0.42,0.57)	0.76	0.79	0.12 (−0.17,0.40)	0.40	0.70
	12 W - Base	−0.09 (−0.59,0.40)	0.71		0.05 (−0.24,0.34)	0.71	
	12 W - 1 W	−0.17 (−0.66,0.33)	0.50		−0.07 (−0.36,0.23)	0.65	
R4	1 W - Base	0.27 (−0.26,0.80)	0.31	0.42	0.15 (−0.16,0.45)	0.33	0.57
	12 W - Base	−0.06 (−0.59,0.47)	0.81		0.02 (−0.30,0.33)	0.91	
	12 W - 1 W	−0.33 (−0.86,0.20)	0.22		−0.13 (−0.45,0.18)	0.40	
Worst	1 W - Base	0.18 (−0.20,0.56)	0.35	0.45	0.18 (−0.04,0.40)	0.10	0.25
	12 W - Base	−0.05 (−0.43,0.33)	0.79		0.06 (−0.16,0.29)	0.57	
	12 W - 1 W	−0.23 (−0.61,0.15)	0.23		−0.12 (−0.34,0.11)	0.29	

The ROIs are mentioned as R1 to R4 along with the area of worst perfusion, measured over the study duration. *P*-values are from repeated measure analysis of variance models. Base = baseline; W = week; Worst = worst perfusion. CI, confidence interval; ROI, region of interest.

**Table 3:** **Summary of changes of all region perfusion responses by treatment and time under cold conditions**

		**Placebo**			**Ambrisentan**		
**Measure**	**Change period**	**Change (95% CI)**	**Change** ***P*** **-value**	**Overall** ***P*** **-value**	**Change (95% CI)**	**Change** ***P*** **-value**	**Overall** ***P*** **-value**
All	1 W - Base	−0.13 (−0.50,0.24)	0.47	0.62	0.07 (−0.15,0.28)	0.52	0.39
	12 W - Base	−0.17 (−0.54,0.20)	0.35		−0.08 (−0.30,0.14)	0.46	
	12 W - 1 W	−0.04 (−0.41,0.33)	0.82		−0.15 (−0.37,0.07)	0.17	
R1	1 W - Base	−0.07 (−0.40,0.25)	0.65	0.65	0.19 (−0.00,0.38)	0.054	0.036
	12 W - Base	−0.15 (−0.48,0.18)	0.35		−0.06 (−0.25,0.13)	0.54	
	12 W - 1 W	−0.08 (−0.41,0.25)	0.64		−0.25 (−0.44,-0.05)	0.015	
R2	1 W - Base	−0.05 (−0.45,0.36)	0.81	0.79	0.07 (−0.17,0.30)	0.56	0.33
	12 W - Base	−0.13 (−0.54,0.27)	0.51		−0.11 (−0.35,0.13)	0.36	
	12 W - 1 W	−0.09 (−0.49,0.32)	0.67		−0.18 (−0.42,0.06)	0.14	
R3	1 W - Base	−0.19 (−0.59,0.21)	0.35	0.54	0.07 (−0.16,0.30)	0.54	0.44
	12 W - Base	−0.19 (−0.59,0.21)	0.33		−0.08 (−0.32,0.16)	0.50	
	12 W - 1 W	−0.01 (−0.41,0.39)	0.98		−0.15 (−0.39,0.09)	0.21	
R4	1 W - Base	−0.22 (−0.69,0.25)	0.36	0.57	−0.05 (−0.32,0.22)	0.70	0.84
	12 W - Base	−0.21 (−0.69,0.26)	0.37		−0.08 (−0.36,0.20)	0.57	
	12 W - 1 W	0.01 (−0.47,0.48)	0.98		−0.03 (−0.31,0.25)	0.84	
Worst	1 W - Base	−0.11 (−0.43,0.20)	0.47	0.41	0.18 (−0.01,0.36)	0.058	0.031
	12 W - Base	−0.21 (−0.53,0.11)	0.19		−0.07 (−0.26,0.12)	0.46	
	12 W - 1 W	−0.10 (−0.41,0.22)	0.54		−0.25 (−0.43,-0.06)	0.012	

The ROIs are mentioned as R1 to R4 along with the area of worst perfusion, measured over the study duration. *P*-values are from repeated measure analysis of variance models. Base = baseline; W = week; Worst = worst perfusion. CI, confidence interval; ROI, region of interest.

Under normal conditions (Table 2), overall, there was a 0.11 pU increase in perfusion in the ambrisentan group as compared to 0.18 pU increase in the placebo group from baseline to week 1 (*P* = 0.41); there was a 0.01 pU decrease in the ambrisentan group as compared to 0.06 pU decrease in the placebo group from baseline to week 12 (*P* = 0.93). There was a 0.12 pU decrease in the ambrisentan group versus 0.24 pU decrease in the placebo group from week 1 to week 12 (*P* = 0.37). Under normal conditions, no significant differences were noted overall (*P* = 0.61), or specifically in the areas of worst perfusion (*P* = 0.25).

Under cold conditions (10°C for two minutes), there was a 0.07 pU increase in the overall flow in the ambrisentan group as compared to 0.13 pU decrease in the placebo group from baseline to week 1 (*P* = 0.52) (Table 3); a 0.08 pU decrease in the overall flow in the ambrisentan group versus 0.17 pU decrease in the placebo group from baseline to week 12 (*P* = 0.46), and a 0.15 pU decrease in the overall flow in the ambrisentan group versus 0.04 pU decrease in the placebo group from week 1 to week 12 (*P* = 0.17). No significant differences were noted under cold conditions (*P* = 0.39). While no significant changes in the areas of worst perfusion were noted between the two groups from baseline to week 12 (*P* = 0.46), on cold challenge, there were significant decreases in perfusion in the ambrisentan group from week 1 to week 12 in R1 (*P* = 0.015) and the regions of worst perfusion (*P* = 0.012).

Table 4 shows the changes in survey responses within each group. Significant improvements in the S-HAQ scores and RCS were seen in the ambrisentan group at week 1 and week 12 relative to baseline, implying better quality of life and less Raynaud attacks. For the S-HAQ scores, there was a 0.12 point decrease (improvement) in the ambrisentan group versus 0.13 in the placebo group from baseline to week 1, 0.20 point decrease in both groups from baseline to week 12, and a 0.09 point decrease in the ambrisentan group versus 0.08 in the placebo group from week 1 to week 12; overall, there was a significant improvement in the S-HAQ scores over time (*P* = 0.005). On the RCS, there was a 0.20 point increase in the ambrisentan group versus a 0.20 point decrease in the placebo group from baseline to week 1, but then a 1.46 point decrease in the ambrisentan group versus a 0.20 point decrease in the placebo group from baseline to week 12, and a 1.66 point decrease in the ambrisentan group versus no change in the placebo group from week 1 to week 12. We did observe a statistically significant improvement in the RCS over time (*P* = 0.001). For the P-VAS scores, there was a 0.47 point decrease (improvement) in the ambrisentan group versus a 0.20 point increase (worsening) in the placebo group from baseline to week 1, a 0.66 point decrease in the ambrisentan group versus 0.80 decrease in the placebo group from baseline to week 12, and a 0.20 point decrease in the ambrisentan group as compared to 1.00 decrease in the placebo group from week 1 to week 12 (*P* = 0.14). Therefore, there was no significant change overall in the P-VAS scores. All patients completed the first two visits. One patient did not complete the third visit and another stopped the study medication after four weeks but subsequently completed the 12 week visit. Both these subjects were in the ambrisentan group (Figure 1).

**Table 4:** **Summary of changes in all survey responses by treatment and time**

		**Placebo**			**Ambrisentan**		
**Measure**	**Change period**	**Change (95% CI)**	**Change** ***P*** **-value**	**Overall** ***P*** **-value**	**Change (95% CI)**	**Change** ***P*** **-value**	**Overall** ***P*** **-value**
S-HAQ	1 W - Base	−0.13 (−0.33,0.08)	0.22	0.14	−0.12 (−0.23,-0.00)	0.048	0.005
	12 W - Base	−0.20 (−0.40,0.00)	0.051		−0.20 (−0.32,-0.08)	0.001	
	12 W - 1 W	−0.08 (−0.28,0.13)	0.45		−0.09 (−0.21,0.03)	0.15	
RCS	1 W - Base	−0.20 (−1.74,1.34)	0.79	0.95	0.20 (−0.69,1.09)	0.65	0.001
	12 W - Base	−0.20 (−1.74,1.34)	0.79		−1.46 (−2.37,-0.55)	0.002	
	12 W - 1 W	−0.00 (−1.54,1.54)	0.99		−1.66 (−2.57,-0.75)	<0.001	
P-VAS	1 W - Base	0.20 (−0.95,1.35)	0.73	0.19	−0.47 (−1.13,0.20)	0.16	0.14
	12 W - Base	−0.80 (−1.95,0.35)	0.17		−0.66 (−1.35,0.02)	0.056	
	12 W - 1 W	−1.00 (−2.15,0.15)	0.087		−0.20 (−0.88,0.49)	0.56	

*P*-values are from repeated measure analysis of variance models. Base = baseline; W = week; CI, confidence interval; P-VAS, Visual Analog Scale for Pain; RCS, Raynaud’s Condition Score; S-HAQ, Scleroderma Health Assessment Questionnaire.

### Adverse events

One patient in the placebo group had a drop in hemoglobin from 11 g/dl to 10 g/dl at one month which returned to 11 g/dl on repeat testing. Another patient was hospitalized for viral pneumonia.

## Discussion

This is the first randomized, double-blind, placebo-controlled study investigating the effect of an ET_A_ receptor antagonist on digital microvascular flow in limited SSc. The purpose of choosing ambrisentan, a selective ET_A_ antagonist (as opposed to bosentan, a dual ET_A_ and ET_B_ antagonist) was its ability to permit the vasodilator effect of endothelin through its interaction with the ET_B_ receptor, while inhibiting its vasoconstrictive effect through its interaction with the ET_A_ receptor. Although the practical implications and advantages (if any) of this selective inhibition are not clear at this time, it was speculated that maintaining the vasodilator effect of endothelin by unimpeded ET_B_ receptor stimulation might have an advantage over bosentan in augmenting digital microvascular flow in SSc patients.

Our results indicated no improvement at week 1 in the mean microvascular blood flow compared to placebo, indicating no demonstrable vasodilatory effect of ambrisentan. Also, at week 12, the differences in mean blood flow did not reach statistical significance, indicating no significant anti-fibro-proliferative effect of ambrisentan either, in the given time frame of three months. We did observe a significant improvement in the S-HAQ score (*P* = 0.005) and the RCS (*P* = 0.001), with no detectable change in the P-VAS score (*P* = 0.14).

The possibility of inter-observer differences was eliminated by having the same investigator (NB) consistently mark the ROIs for each patient and comparing to corresponding images from previous visits. Patients were periodically counseled to ensure medication compliance.

Based on data derived from our preliminary study [11], we defined a minimal clinically important difference (MCID) between the two arms. A small sample size is unlikely to be the primary cause of non-significance in the study since we indeed observed worsening perfusion in some regions over time. If these trends remained, it is unlikely that an increase in sample size would have led to a change in the conclusion that ambrisentan fails to induce clinically meaningful improvement in perfusion. Therefore, we conclude that a three-month course of ambrisentan is ineffective in improving digital microvascular flow in patients with limited SSc.

In a recent open label study, bosentan was used in SSc patients with PAH along with nifedipine [17]. Although digital blood flow improved, symptoms of RP were not decreased. Moreover, it was difficult to assess the relative contribution of bosentan on digital blood flow, as the patients also received nifedipine.

Under physiologic conditions, cutaneous blood flow is extremely variable and may be affected by a number of intrinsic and extrinsic factors that vary over time, for example, systemic blood pressure, body temperature, ambient temperature, stress level, hemoglobin level, food intake, smoking status, medications, and so on. Many other unidentified factors are also probably involved in cutaneous vasoregulation. Digital blood flow also varies considerably between subjects. Because of these variables, accurate evaluation of digital microvascular flow is difficult. Consequently, a clinically meaningful appraisal of the effect of vasoactive therapy on digital blood flow is extremely challenging.

In addition to the physiological variability discussed above, scleroderma patients have some additional challenges that affect reliable interpretation of LDPI measurements. Mapping the ROIs is difficult in some SSc patients because of contractures in their hands and fingers. Moreover, the depth of penetration of the laser beam could vary according to the degree of skin thickening at the ROIs. Seventy-five percent of our patients were white and the rest were non-white; this may also be relevant, as the melanin content in the skin can influence the passage of laser beams and perfusion measures. Furthermore, as progressive microvascular fibrosis and small blood vessel rarefaction is known to occur over time, the duration of SSc diagnosis may affect digital blood flow. Although, it is assumed that the variables that affect digital blood flow should equally affect ambrisentan and placebo arms, variability due to chance cannot be excluded with small sample sizes. Nevertheless, in spite of all of the limitations mentioned above, we used LDPI in our study, as it has several advantages over most other existing methods in objectively assessing digital microvascular flow, and has hence become an established technology in the assessment of digital micro-circulation in scleroderma [10-18].

The results of our study indicate that ambrisentan, an ET_A_ receptor antagonist, does not significantly impact digital microvascular flow in SSc patients. Although digital arterioles are similar to pulmonary arterioles both structurally and in size, there may be unknown differences that could possibly explain this differential vaso-modulator response to ambrisentan, including certain intrinsic variations in the blood vessels that are presently unknown, or differences in the local micro- milieu. Thus, ACE-inhibitors are effective and life-saving in an SSc renal crisis, establishing an important role of the renin-angiotensin system in the renal microvasculopathy in SSc; yet, these agents are ineffective in the treatment of SSc associated RP [29].

It has been suggested by prior studies that although bosentan may be effective in preventing ischemic digital ulcers, it does not alleviate SSc-associated RP and hand pain [2,9]. This may indicate that digital blood flow and ischemic digital ulceration and necrosis are possibly mediated by overlapping but not necessarily congruent pathways or mediators; conceivably, both of these pathways are dysregulated in SSc, the latter being partially controlled by endothelin, but not necessarily the former. Possibly other potential mediators of microvascular flow such as catecholamines, angiotensin II, prostanoids, and nitric oxide may play a more prominent role than endothelin in the regulation of digital microcirculation in SSc.

The discrepancy between the lack of improvement in blood flow and improvement in the RCS and S-HAQ scores remains unexplained, but suggests that these subjective measures of symptomatic improvement of Raynaud phenomena and of functional status may not be entirely mediated through reversal of digital ischemia, but possibly through other factors, such as improvement in hand function and overall quality of life. This decoupling might be perceived to hint at some off target effects of ambrisentan not involving vasodilatory or anti-proliferative pathways.

## Conclusions

Continuous use of ambrisentan for three months did not demonstrate any evidence of improvement of digital microperfusion in limited SSc patients although patient oriented outcomes were quite favorable. Larger, prospective trials are needed to validate our results.

## Additional files

Additional file 1:
**Scleroderma-HAQ-Disability Index.**
Additional file 2:
**Raynaud's Condition Score.**
Additional file 3:
**Pain-Visual Analog Scale.**

## Abbreviations

ALT: alanine aminotransferase
AST: aspartate aminotransferase
ET-1: endothelin-1
ET_A_: endothelin A
ET_B_: endothelin B
IUD: intrauterine device
LDPI: laser Doppler perfusion imaging
PAH: pulmonary arterial hypertension
pU: perfusion unit
P-VAS: pain-visual analog scale
RCS: Raynaud condition score
REDCap: research electronic data capture
ROI: region of interest
RP: Raynaud phenomenon
SD: standard deviation
S-HAQ: Scleroderma-health assessment questionnaire
SSc: systemic sclerosis
