# Supplementary material for: Evaluation of the effect of ambrisentan on digital microvascular flow in patients with systemic sclerosis using laser Doppler perfusion imaging: a 12-week randomized double-blind placebo controlled trial
Source: Arthritis Res Ther. 2015 Mar 5;17(1):44. doi: 10.1186/s13075-015-0558-9 (PMC4384235; doi:10.1186/s13075-015-0558-9)
Supplement: Additional file 1: — Scleroderma-HAQ-Disability Index. [file 13075_2015_558_MOESM1_ESM.doc]

**Additional file 1**

**Disability Index**

**(Scleroderma Health Assessment Questionnaire)**

**Disability Index**

**(Scleroderma Health Assessment Questionnaire)**

**Visit number: 1 2 3**

**Date: ______/______/______**

**Research ID number: ____________________**

Please check the answer that best describes your usual abilities in the past 7 days.

| | **Are you able to:** | **Without**  **any Difficulty** | **With Some**  **Difficulty** | **With Much**  **Difficulty** | **Unable**  **to do** | ***Office***  ***Use*** | | --- | --- | --- | --- | --- | --- | | Dress yourself, including tying  shoelaces & doing buttons?  Shampoo your hair? |  |  |  |  | **Dressing &**  **Grooming** | | Stand up from an armless  straight chair?  Get in and out of bed? |  |  |  |  | **Arising** | | Cut your meat?  Lift a full glass to your mouth?  Open a new milk carton? |  |  |  |  | Eating | | Walk outdoors on flat ground?  Climb up five stairs? |  |  |  |  | **Walking** | | Wash and dry your entire body  Take a bath  Get on and off the toilet |  |  |  |  | **Hygiene** | | **Are you able to:** | **Without**  **any Difficulty** | **With Some**  **Difficulty** | **With Much**  **Difficulty** | **Unable**  **to do** | ***Office***  ***Use*** | | Reach and get down a heavy  Object (such as bag of sugar,  or a large book) from just over  your head?  Bend down and pick up  clothing off the floor ? |  |  |  |  | **Reach** | | Open car doors?  Open jars that have been  Previously opened?  Turn taps on and off? |  |  |  |  | **Grip** | | Run errands and shop?  Get in and out of a car?  Do household cleaning such  as vacuuming or sweeping? |  |  |  |  | **Activity** | | **FOR OFFICE USE:**  Transfer the worst score | Without any  difficulty= 0 | With some  Difficulty=1 | With much  Difficulty=2 | Unable to  Do=3 | TOTAL= | |
| --- | --- | --- | --- | --- | --- | --- | --- | --- | --- | --- | --- | --- | --- | --- | --- | --- | --- | --- | --- | --- | --- | --- | --- | --- | --- | --- | --- | --- | --- | --- | --- | --- | --- | --- | --- | --- | --- | --- | --- | --- | --- | --- | --- | --- | --- | --- | --- | --- | --- | --- | --- | --- | --- | --- | --- | --- | --- | --- | --- | --- | --- | --- | --- | --- | --- | --- |
