# Supplementary material for: Evaluation of the effect of ambrisentan on digital microvascular flow in patients with systemic sclerosis using laser Doppler perfusion imaging: a 12-week randomized double-blind placebo controlled trial
Source: Arthritis Res Ther. 2015 Mar 5;17(1):44. doi: 10.1186/s13075-015-0558-9 (PMC4384235; doi:10.1186/s13075-015-0558-9)
Supplement: Additional file 2: — Raynaud's Condition Score. [file 13075_2015_558_MOESM2_ESM.doc]

**Additional file 2**

**Raynaud’s Condition Score**

**The Raynaud’s Condition Score is a daily self-assessment of Raynaud’s phenomenon activity using a 0–10 ordinal scale. The Raynaud’s Condition Score incorporates the cumulative daily frequency, duration, severity, and impact of Raynaud’s attacks.**

**Raynaud’s Condition Score**

**Visit number: 1 2 3**

**Date: ______/______/______**

**Research ID number: ____________________**

**Instructions:** The Raynaud’s Condition Score is your rating of how much difficulty you had with your Raynaud’s TODAY. Consider how many Raynaud’s attacks you had and how long they lasted. Consider how much pain, numbness, or other symptoms the Raynaud’s caused in your fingers (including painful sores) and how much the Raynaud’s ALONE affected the use of your hands today. Please circle the number that best describes your response.

**None Mild Moderate Severe Worst possible**

**0 1 2 3 4 5 6 7 8 9 10**
