# Supplementary material for: Evaluation of the effect of ambrisentan on digital microvascular flow in patients with systemic sclerosis using laser Doppler perfusion imaging: a 12-week randomized double-blind placebo controlled trial
Source: Arthritis Res Ther. 2015 Mar 5;17(1):44. doi: 10.1186/s13075-015-0558-9 (PMC4384235; doi:10.1186/s13075-015-0558-9)
Supplement: Additional file 3: — Pain-Visual Analog Scale. [file 13075_2015_558_MOESM3_ESM.doc]

**Additional file 3**

**Pain – Visual Analog Scale**

**Pain – Visual Analog Scale**

**Visit number: 1 2 3**

**Date: ______/______/______**

**Research ID number: ____________________**

**Instructions:** Please circle the number that best describes your **typical** or **average** pain.

**None Mild Moderate Severe Worst possible**

**0 1 2 3 4 5 6 7 8 9 10**
